# Effect of Low Molecular Weight Oxidized Materials and Nitrogen Groups on Adhesive Joints of Polypropylene Treated by a Cold Atmospheric Plasma Jet

**DOI:** 10.3390/polym13244396

**Published:** 2021-12-15

**Authors:** Kateřina Polášková, Miloš Klíma, Zdeňka Jeníková, Lucie Blahová, Lenka Zajíčková

**Affiliations:** 1 Department of Theoretical and Experimental Electrical Engineering, Faculty of Electrical Engineering and Communication, Brno University of Technology, Technická 12, CZ-61600 Brno, Czech Republic; polaskova.katerina@mail.muni.cz (K.P.); klimam@vut.cz (M.K.); 2 Department of Dept Condensed Matter Physics, Faculty of Science, Masaryk University, Kotlářská 2, CZ-61137 Brno, Czech Republic; 3 Department of Materials Engineering, Faculty of Mechanical Engineering, Czech Technical University in Prague, Karlovo Náměstí 13, CZ-12000 Prague, Czech Republic; Zdenka.Jenikova@fs.cvut.cz; 4 Central European Institute of Technology–CEITEC, Brno University of Technology, Purkyňova 123, CZ-61200 Brno, Czech Republic; Lucie.Blahova@ceitec.vutbr.cz

**Keywords:** cold atmospheric plasma, plasma treatment, adhesion, polypropylene, X-ray photoelectron spectroscopy, water contact angle

## Abstract

Polypropylene is a typical representative of synthetic polymers that, for many applications including adhesive joints, requires an increase in wettability and chemical surface reactivity. Plasma processing offers efficient methods for such surface modifications. A particular disadvantage of the plasma jets can be the small plasma area. Here, we present a cold atmospheric plasma radio-frequency slit jet developed with a width of 150 mm applied to polypropylene plasma treatment in Ar, Ar/O${}_{2}$ and Ar/N${}_{2}$ We identified two main parameters influencing the tensile strength of adhesive joints mediated by epoxy adhesive DP 190, nitrogen content, and the amount of low molecular weight oxidized materials (LMWOMs). Nitrogen functional groups promoted adhesion between epoxy adhesive DP 190 and the PP by taking part in the curing process. LMWOMs formed a weak boundary layer, inhibiting adhesion by inducing a cohesive failure of the joint. A trade off between these two parameters determined the optimized conditions at which the strength of the adhesive joint increased 4.5 times. Higher adhesion strength was previously observed when using a translational plasma gliding arc plasma jet with higher plasma gas temperatures, resulting in better cross linking of polymer chains caused by local PP melting.

## 1. Introduction

Plasma treatment of polymers is a well-established technique used to increase wettability and, subsequently, the adhesion of a polymer surface [1,2,3]. Over the years, a wide variety of plasma source types, each one with different advantages and drawbacks, has been applied to the modification of various synthetic polymers (e.g., polypropylene, polyethylene, and polytetrafluoroethylene). As the topic of plasma treatment is quite broad, we will focus only on the results previously achieved for the synthetic polymer utilized in this study: polypropylene.

Polypropylene (PP) is a cheap, versatile, and fully recyclable thermoplastic polymer with many beneficial properties such as low density, resistance to corrosion, or high thermal and chemical stability [4,5]. It is the most widely used thermoplastic, with applications ranging from food packaging to fabrics and plastic tools manufacturing to automotive industry [6]. PP has intrinsically low surface free energy (27 J m${}^{-2}$ [7]) and chemical reactivity that, while advantageous in some applications, prove to be limiting in other ones (e.g., a bond between the PP and a standard adhesive). A modification of PP surface properties (wettability and adhesion) is required in the second case. The standard method used to increase surface energy is a chemical treatment by solvent-based adhesion promoters (primers) [8]. However, the usage of primers has two significant drawbacks. The negative effect on the environment is due to the primers’ toxicity and the high time demands as they have to be applied manually onto a polymer surface. Out of the possible environmentally friendly and automated alternatives, such as flame treatment [9,10] and UV irradiation [11], plasma treatment appears to be the most promising one [12].

Throughout the years, several different non-thermal plasma discharges were used to treat polypropylene, namely low pressure [13,14,15], corona and dielectric barrier discharges (DBD) [16], and cold atmospheric plasma (CAP) jets [17,18]. The adhesion between a plasma-treated polymer and an adhesive is a complicated process influenced primarily by a combination of three main parameters, functional groups composition and concentration, surface morphology, and the amount of the light molecular weight oxidized matter (LMWOM). The contribution of each of the parameters can differ from system to system. For example, in a system of low-pressure plasma-treated PP sheets bonded together using a two-component epoxy adhesive studied by Mandolfino et al. [14,15], an increase in adhesion was directly proportional to the chemical insertion of polar groups, mainly hydroxy groups C–OH. Changes in roughness did not affect the resulting adhesion. On the other hand, in a bond between PP treated by a CAP jet and acrylate-based adhesion PSA tapes studied by Kehrer et al. [17], changes in surface nanostructure were identified as the main reason behind the observed adhesion differences. As a general rule, adhesion increases with the increasing number of polar functionalities (providing more reactive bonds available to react with the adhesive) and higher surface roughness (increasing the area available for the adhesive–polymer interface).

Most of the functional groups attached during the plasma treatment exist on the top of the treated surface as soluble LMWOM and shorter oxidized PP chains weakly attached to the bulk [18]. The influence of LMWOM on the adhesion is not as straightforward as in the case of chemical composition or roughness. They usually act as a so-called “weak boundary layer” between the adhesive and the polymer surface, reducing final adhesion. In some cases, LMWOMs can be incorporated into the adhesive, resulting in adhesion enhancement [19]. Even though the adhesion improving effects of plasma treatment have been investigated for several decades, detailed studies correlating exact surface characteristics with adhesion results are still rare, especially for Ar-based CAP jets.

CAP jets are versatile devices because the low gas temperature enables heat-sensitive materials processing, and the plasma plume extends several centimeters into an open space enabling the processing of complex material shapes at variable distances. Their particular disadvantage can be the small plasma area. If the treatment of a wider area is required and the jet movement is limited, a jet array must be constructed. This naturally multiplies the system’s running cost. The construction of a jet array is also not a straightforward process, as the interaction of individual plasma plumes can have a significant effect on the treatment uniformity [20].

For the first time, we present a radio-frequency plasma slit jet (PSJ), a CAP developed in the width of 150 mm and demonstrate its performance on the polypropylene plasma treatment in Ar, Ar/O${}_{2}$, and Ar/N${}_{2}$. The advantage of PSJ over the other atmospheric pressure plasma jets lies in a wide active plasma interacting with surfaces. We focused on identifying the parameters necessary for the tensile strength of adhesive joints between PP and Al mediated by epoxy adhesive DP 190. Therefore, we analyzed the functional groups at the PP surface, the presence of LMWOMs, water contact angle, and surface morphology. We optimized the jet working parameters (power, Ar, and N${}_{2}$ flow rate) for the most promising gas feed, Ar/N${}_{2}$, to achieve the maximum tensile strength (4.5 times increased). The improved tensile strength is explained by nitrogen groups taking part in the adhesive curing process. However, we observed that the nitrogen functionalities are not incorporated in medium or longer-length polypropylene chains but only in LMWOMs. Thus, our original finding is the correlation between higher adhesion and higher nitrogen functional group content, supposing that the amount of LMWOMs is kept at a similar level. A unique conclusion can be drawn by taking into account our previously published results on the PP treatment with a translational plasma, gliding-arc plasma jet [21]. The higher adhesion strength after PP treatment by the gliding arc is caused by a higher gas temperature that results in local melting of the topmost polymer layer, bonding it better to the PP bulk.

## 2. Materials and Methods

### 2.1. Polypropylene Samples Used for Plasma Treatment

Polypropylene, in the form of 5 mm thick sheets, was provided by the company Omniplast, Podolí u Brna, Czech Republic under the product name PP-H Natural. The sheets were produced by the extrusion of a PP granulate using three rollers. The bottom of the PP sheet was in contact with the rollers and the top side was free. Even though the rollers were smooth and rotated freely, the bottom side became rougher than the top one. The bottom side of PP sheets was covered by a protective foil directly on a conveyor belt while the top side was covered manually after the extrusion process.

The less rough top side of PP was chosen for the experiments. PP sheets were cut into stripes of two different sizes: 100×25 mm${}^{2}$ for water contact angle (WCA) and tensile strength tests, and 10×12.5 mm${}^{2}$ for X-ray photoelectron spectroscopy (XPS) and confocal microscopy. The protective foil was removed from the treated PP side before the experiments. The cleaning procedure was designed to remove glue residues as follows:

1. Wiping PP surface with a cleanroom wipe soaked in isopropanol (100%, Lach-Ner, Neratovice, Czech Republic);
2. Wiping PP surface with a cleanroom wipe soaked in cyclohexane (99.94%, Lach-Ner, Neratovice, Czech Republic);
3. Immersion of the PP in isopropanol (IPA) and ultrasonication for 10 min;
4. Wiping the surface with an IPA-soaked cleanroom wipe.

At least ten subsequent wipes were carried out in the steps one and two.

### 2.2. RF Plasma Slit Jet Set-Up

**Figure 1:**
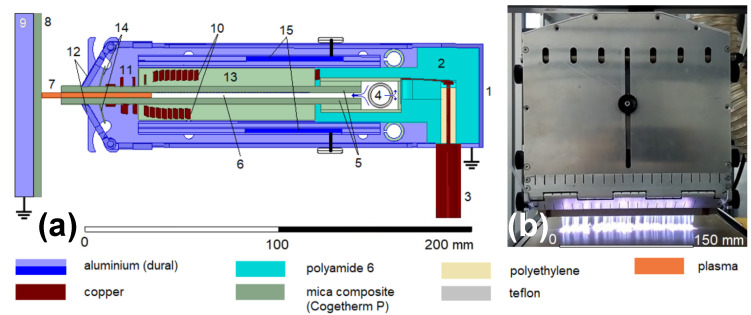
(**a**) Schematic drawing of the RF plasma slit jet (side view): 1—aluminum cover/shielding; 2—load-bearing element; 3—coaxial cable; 4—Ar flow homogenization region; 5—mica composite slit body; 6—slit; 7—plasma region; 8—sample; 9—grounded metal desk; 10—resonance coil; 11—high-voltage radio-frequency electrodes; 12—system of movable grounded electrodes; 13—dielectric plates of an electrical breakdown limiter; 14—plates supporting the coil turns; 15—conductive plates of resonance matching circuit. (**b**) Front view of RF plasma slit jet running in pure Ar with a mica composite sheet as a substrate.

The RF plasma slit jet (PSJ) was constructed with a matching element integrated in the plasma jet as a periodic deceleration structure consisting of an inductor with specially designed geometry and winding. The jet body made of the mica composite was placed inside the coil (Figure 1). Both were situated inside the tunable cavity made up of the metal cover/shielding and conductive plates that were used as the resonance matching circuit. Therefore, the RF plasma slit jet can be connected directly to the RF generator by using a coaxial cable and no separate matching unit is needed. A uniform gas flow at all parts of the slit exit is ensured by creating random turbulences at the upper part of the jet body using a tube with small evenly distributed holes facing the top-most metal cover. As the turbulently mixed working gas starts flowing downwards through the slit, it homogenizes, resulting in a uniform flow at the slit exit. Hence, if the gas mixtures are used, it is necessary to mix the individual gases before they enter the RF plasma slit jet.

The working frequency of the RF plasma slit jet was 13.56 MHz, and RF power ranged from 300 to 600 W. Three gas feeds were studied: Ar, Ar/O${}_{2}$, and Ar/N${}_{2}$. The Ar flow rate was from 50 to 100 slm. The added O${}_{2}$ flow rate was 1 slm while the added N${}_{2}$ flow rate varied between 1 and 4 slm. The distance between the slit exit and the substrate was 5 or 10 mm. PP samples were placed on a conveyor belt moving at the speed 22 or 100 mm/s. The conditions used to compare the influence of the different gas feeds and treatment times are summarized in Table 1.

### 2.3. Methods Used for Surface Characterization

The contact angles of demineralized water and diiodomethane (≥99.4%, VWR Chemicals) were measured immediately and 24 h after the plasma treatment by the sessile drop method using the See System (Advex Instruments, Brno, Czech Republic). Each measurement set consisted of 12 water droplets (3 μL in volume), and the contact angle was determined by using the three-point algorithm implemented in the software See System 7.0 (Advex Instruments, Brno, Czech Republic). The average contact angle values were calculated as truncated means with the lowest and highest contact angles omitted. The surface free energy (SFE) was determined by the Owens–Wendt–Rabel–Kaelble (OWRK) method [22]. Demineralized water was used for polar and diiodomethane for the dispersive part of SFE.

Structural changes of the PP surfaces were visualized using a confocal laser scanning microscope LEXT OLS400 (Olympus, Shinjuku, Tokyo, Japan). The microscope can achieve the resolution of 12 μm when utilizing a laser with wavelength 400 nm. The samples were examined at 100× magnification. Roughness was evaluated by using the Gwyddion software developed by Czech Metrology institute, Brno, Czech Republic [23].

Surface chemical composition was characterized by XPS using the Axis Supra (Kratos Analytical, Manchester, United Kingdom) spectrometer with the monochromatic Kα radiation. The samples were analyzed at two different times: immediately and 24 h after the plasma modification. The time interval between the treatment and the transfer of samples into the XPS vacuum system was 30 min maximum. The survey spectrum was measured using the pass energy of 80 eV with the step of 1 eV. The pass energy of 20 eV, the step 0.1 eV, and five-time accumulation were used for the high resolution measurements. All spectra were acquired in at least two different places (to detect potential treatment inhomogeneity) in the so-called slot mode in which the electrons were collected from an area of 700×300 μm^2^. Differential charging of the surface was prevented by charge neutralization. High-resolution spectra were fitted by the CASA XPS software, Casa Software Ltd, Teignmouth, UK, after the subtraction of Tougaard-type background. The details of the fitting are provided in the Section 3.1. Binding energy was calibrated by shifting the C–C/CH${}_{x}$ peak to the energy 285 eV.

The presence of LMWOMs was analyzed by a water-washing test. The samples were dipped into demineralized water for 30 s directly after the plasma treatment and subsequently dried by a nitrogen gas stream. Surface analyses were carried out immediately after the drying step.

The tensile strength of the adhesive joint between PP and aluminum stripes (25×100×5 mm${}^{3}$ and 25×100×1.5 mm${}^{3}$, respectively) created with the two-component epoxy adhesive DP190 (3M, Maplewood, Minnesota, USA) was measured according to the standard (668510) ČSN EN 1465 [24]. The aluminum stripe was mechanically roughed to ensure excellent adhesion to the epoxy glue. The dimension of the joint part was 12.5×25 mm${}^{2}$. For each treatment condition, a set of 10 samples was prepared. The preparation of the PP-epoxy-Al joint was carried out one day after the plasma treatment. The joints were weighted down by the weight of 0.7 kg per sample for the next 24 h. The tests were carried out after at least seven days to ensure curing of the adhesive. The free ends of the stripes were gripped by claws of the ZD 10 (W + B) tensile tester and pulled away with the velocity of 20 mm/min.

### 2.4. Plasma Diagnostics

Optical emission spectra were recorded by Andor spectrometer model Shamrock 500i coupled to CCD Andor Newton detector (EMCCD DU971P_BV) both manufactured by Oxford Instruments, Abingdon-on-Thames, UK. Overview spectra were measured over a wavelength range 300–900 nm with a grating of 600 L/mm. Exposure time was set to 0.5 s. Therefore, any phenomena with shorter effective time were time-averaged in all measurements. Spectra were acquired without any complex optic system; therefore, taking into account the distance of optical fiber from discharge and acceptance angle, they were considered to be spatially averaged. Atomic lines and molecular bands in measured emission spectra were identified according to the literature [25].

## 3. Results and Discussion

### 3.1. Surface Chemistry of Plasma-Treated PP

All the applied treatment conditions resulted in the incorporation of oxygen and nitrogen functional groups onto the treated PP surface (Table 2). The concentration of oxygen functional groups was always higher than the concentration of nitrogen groups due to oxygen gas species (mainly O and OH radicals) having significantly higher reactivity with the PP chains [26]. For the samples modified at 100 mm/s with two passes, the oxygen content was 10–11 at.% (samples denoted with *f* in Table 2). Nitrogen uptake ranged from trace amounts (samples modified using Ar and Ar/O${}_{2}$ gas feeds) to 1.6 at.% (the sample treated in Ar/N${}_{2}$ gas mixture at the optimized working conditions). By lowering the movement speed to 22 mm/s and increasing the number of passes to ten (samples marked with *s* in Table 2), we further increased oxygen concentration to 20–27 at.% and nitrogen concentration up to 8 at.%.

The effect of the gas feed composition on the overall concentration of functional groups attached to the treated PP samples was observable only at higher treatment times (samples denoted with *s*). Of the three tested gas feeds, the PP surface treated in the Ar/O${}_{2}$ gas mixture contained the lowest concentration of functional groups. The lower efficiency of the Ar/O${}_{2}$ plasma treatment was most likely due to the electronegative character of oxygen plasma. Electrons sustaining the discharge were lost in attachment processes, resulting in the Ar/O${}_{2}$ plasma having a lower electron density than a pure Ar discharge [27]. The lower electron density resulted in lower reactive species concentrations as the reactive species are direct or indirect products of electron impact reactions [28]. Although the addition of N${}_{2}$ into the Ar plasma also reduces electron density [29], the decrease in the number of functional groups attached to the PP surface was minor due to the presence of the Ar/N${}_{2}$ postdischarge (also known as afterglow). Nitrogen afterglow is a diffuse region of a weakly ionized or neutral medium that is no longer considered plasma. In this region, reaction kinetics are no longer driven by electrons but by nitrogen species, mainly nitrogen atoms N and nitrogen metastables N${}_{2}$(A) [29,30]. Nitrogen atoms N, the most abundant species in the afterglow [31], can subsequently react with PP [26] resulting in the attachment of nitrogen functional groups. To summarize, the higher concentration of N in the Ar/N${}_{2}$ postdischarge caused an increase in the probability of the reaction between N atoms and PP, resulting in higher nitrogen uptake on treated PP surface. In contrast, oxygen uptake was lower in the Ar/N${}_{2}$ discharge as the lower plasma electron density resulted in a decrease in the concentration of reactive oxygen species. This behavior is reflected in the atomic constitution of the surface of the ArN-*s* sample.

**Figure 2:**
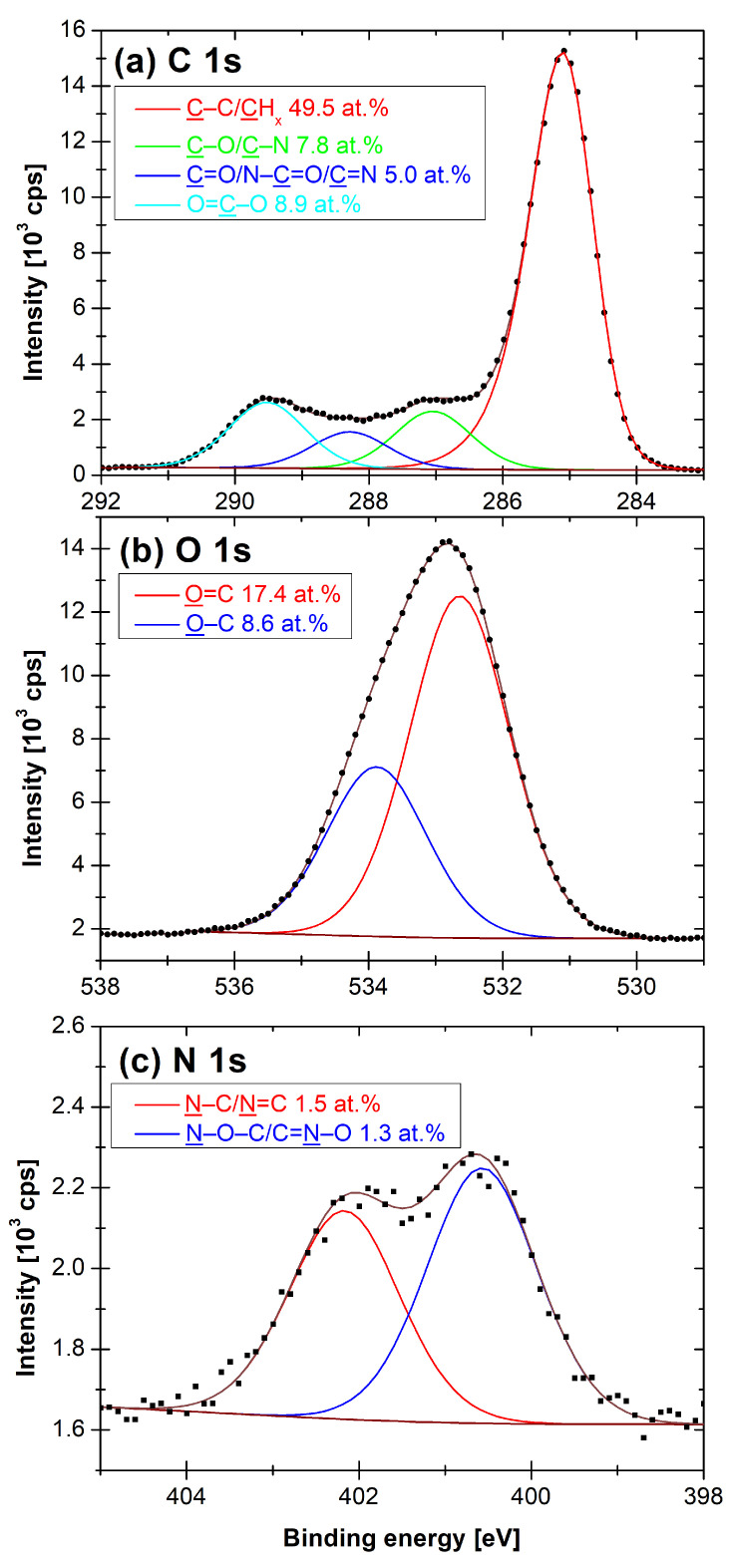
A fitting of (**a**) C 1s, (**b**) O 1s and (**c**) N 1s high-resolution XPS spectra of the Ar-*s* sample.

The assessment of carbon bonds on the PP surface was based on fitting the XPS C 1s signal, as described in our previous article [21]. The four components of C 1s (Figure 2a) were the following: C–C/CH${}_{x}$ (285.0 eV), C–O/–C–N (287.0 ± 0.1) eV, C=O/N–C=O/N=C (288.2 ± 0.1) eV, and O=C–O (289.5 ± 0.1) eV. The C–C/CH${}_{x}$ asymmetric peak shape was derived from the spectrum of pure PP. Its full width at half maximum (FWHM) was (1.05 ± 0.02) eV. The other three components were G–L shaped with the fixed G–L percentage of 30%, G–L(30) and had an FWHM of (1.30 ± 0.02) eV. Both, O 1s and N 1s, high-resolution spectra (Figure 2b,c) were fitted by two G–L(30) components. The positions and bonds were assigned according to the available literature [32,33,34]. The O 1s environment components were O=C (532.8 ± 0.1) eV and O–C (534.1 ± 0.1) eV. The FWHM of O 1s components was (1.78 ± 0.03) eV. The N 1s environment was composed of N–C/N=C (400.6 ± 0.1) eV and N–O–C/C=N–O (402.1 ± 0.1) eV peaks. The FWHM of N 1s components was (1.73 ± 0.05) eV.

**Figure 3:**
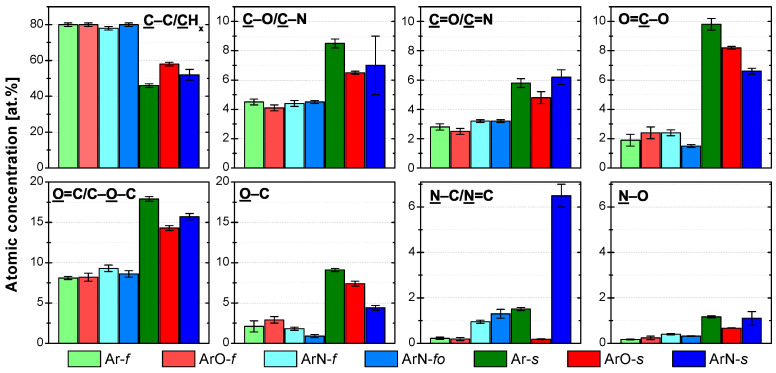
XPS C 1s, O 1s, and N 1s high-resolution spectrum components and their atomic concentrations for the plasma-treated PP samples. Spectra were measured right after the plasma treatment.

For all the analyzed samples, the agreement between the concentration of the C1s environment components (Figure 3) and the measured atomic percentages (Table 2) was good. At the higher treatment speed of 100 mm/s (samples denoted with *f*), the effect of plasma gas composition on the functional groups’ concentration was minor. The only notable difference was the higher number of amine and imine (N–C/N=C) groups at the PP surfaces treated in the Ar/N${}_{2}$ plasma. The increase was due to the reactive nitrogen species-rich afterglow interacting with the PP surface. At 22 mm/s (samples denoted with *s*), the functional group concentrations varied with the gas feed. In the following discussion, sample Ar-*s* (PP treated in pure Ar PSJ) is taken as a base for the comparison. The surface of PP treated in Ar/O${}_{2}$ plasma (sample ArO-*s*) contained primarily oxygen functional groups for which its concentration ratio was similar to the Ar-*s* sample. The number of nitrogen functional groups was much lower at the ArO-*s* surface, and most of them were in an oxidized state. In contrast, for the Ar-*s* sample, the concentration of oxidized and non-oxidized nitrogen functional groups was similar. The low concentration of nitrogen functionalities on the ArO-*s* surface can be explained by how the gases were introduced into the discharge. Oxygen gas was introduced directly into the device intermixed with the main Ar flow. Therefore, most of the energy stored in the plasma—in the form of electrons, argon metastables, etc.—was used to create reactive oxygen species (O, O${}_{2}^{*}$, and O${}_{3}$). Nitrogen was introduced into the jet further downstream as an impurity coming from the surrounding atmosphere. In this region, it is reasonable to assume a low concentration of energetic species as the majority had been used previously in the reactions with oxygen. As a result, only a handful of reactive nitrogen gas species are generated; hence, the low number of nitrogen functional groups attached to the treated PP surface. The oxygen-rich plasma environment then causes their predominant oxidation state.

The addition of N${}_{2}$ had a much larger impact on the concentration of functional groups attached to the treated PP surface (sample ArN-*s* in Figure 3) than the addition of O${}_{2}$. Not only the concentration of nitrogen functionalities increase, as one would expect, but the addition of N${}_{2}$ influenced the composition of oxygen functionalities, mainly the O–C bonds, as well. As the majority of the nitrogen functionalities is present in the form of amine or imine (N–C/N=C) groups, and the concentration of O–C (respectively, O=C–O) bonds decreased, it appears that nitrogen (more abundant) and oxygen (more reactive [26]) gas species compete for mainly the carbon radicals. Considering a PP chain backbone scission, the changes in the functional group concentrations can be explained by two fundamentally different reaction pathways:

1. Nitrogen atoms react with the PP alkyl radicals;

**Figure:**
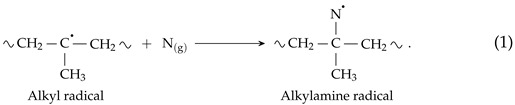
2. Nitrogen atoms react with carbonyl radicals (created by the abstraction of H from the aldehyde groups (3), products of the fi-scission of secondary alkoxy radicals (2)), terminating the PP chain by the amide group (4).

**Figure:**
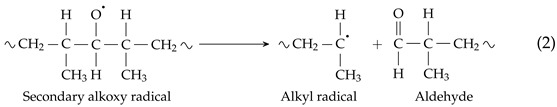

**Figure:**
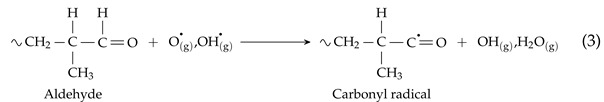

**Figure:**
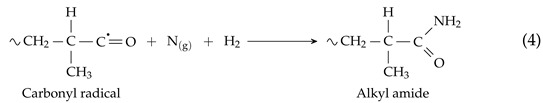

In reaction (1), nitrogen ends up using the carbon radical created by the abstraction of H atoms from the PP backbone, limiting the number of sites where alkoxy radicals (the primary source of the chain backbone scission) could be formed. As a result, the ablation of PP and the formation of low molecular weight oxidized materials (LMWOMs) are reduced. On the other hand, in the reaction pathway (2)–(4), the nitrogen functional group is attached to the end of the ablated shorter PP chain, meaning that it is a part of low molecular weight oxidized materials (LMWOMs). Regarding reactivity, mid-chain alkyl radicals reactivity is much lower than the reactivity of the end-chains radical, making the second reaction pathway more likely. The results of water washing experiments, discussed in detail in Section 3.3, show that most of the nitrogen functionalities are a part of LMWOMs, confirming the reactivity-based assumption. To summarize the effect of the addition of N${}_{2}$ to the Ar gas feed on the functional group composition, the observed decrease in the concentrations of O–C and O=C–C environments was not caused by the lower degree of the PP chain scission but by a portion of the carboxyl terminating groups being replaced with amides.

**Figure 4:**
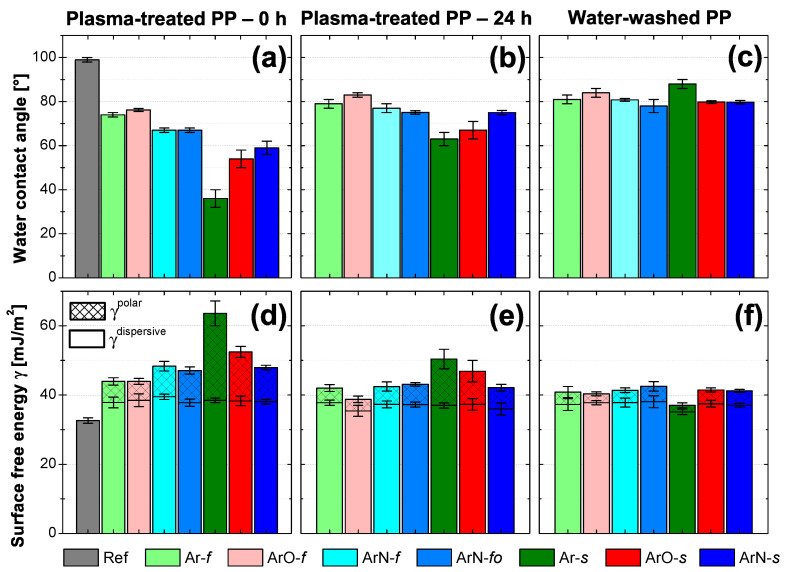
Water contact angle of a sessile droplet measured (**a**) right after treatment (0 h), (**b**) one day after treatment (24 h), and (**c**) after being submerged into demineralized water (water washed); surface free energy divided into the polar and dispersive part determined from contact angles measured (**d**) right after treatment (0 h), (**e**) one day after treatment (24 h), and (**f**) after being submerged into demineralized water (water-washed).

**Figure 5:**
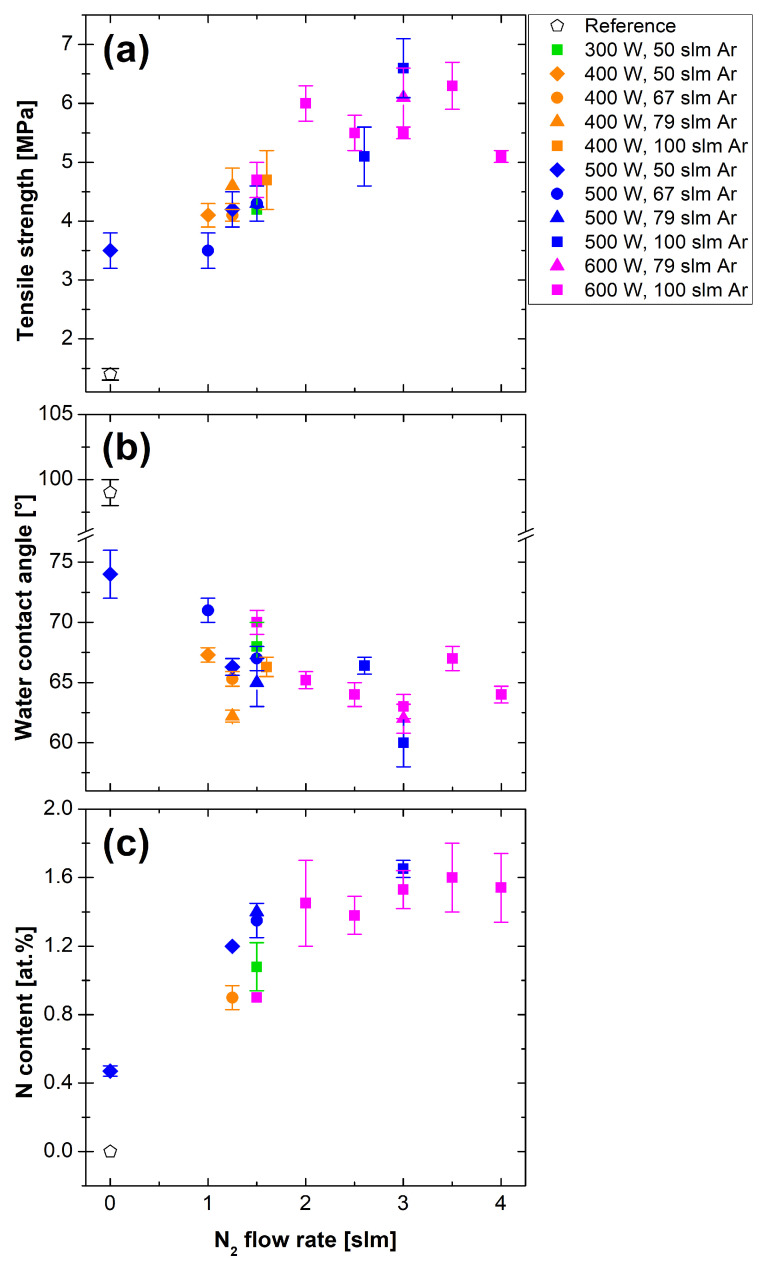
Dependence of the (**a**) tensile strength, (**b**) water contact, angle and (**c**) nitrogen content of the plasma-treated PP on the nitrogen working gas flow rate.

**Figure 6:**
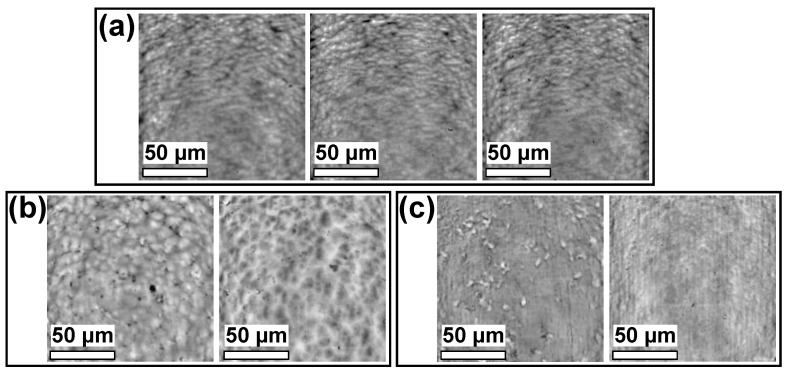
Confocal microscope images for 100× magnification. From left to right: (**a**) reference and plasma-treated and water-washed PP (ArN-*s*); (**b**) PP and epoxy adhesive surface treated at the higher treatment speed (ArN-fo) after the tensile strength test and (**c**) PP and epoxy adhesive surface treated at the lower treatment speed (Ar-*s*) after the tensile strength test.

As shown in Figure 4a,d, the plasma treatment resulted in a decrease in water contact angles (WCA) and an increase in the surface free energy (SFE). Water contact angle measurement can be affected by both the chemical composition and the structure of the treated surface. In our case, the presence of the LMWOMs is another factor that had to be taken into account. LMWOMs can be dissolved by polar solvents such as water influencing the measured WCA. For the samples treated at 100 mm/s, surface micro-structure remained unchanged as no difference in the roughness before and after the treatment was observed (Table 2). Therefore, measured WCAs differences mostly came from the changes in surface chemistry. WCAs of the PP surfaces treated in the Ar and Ar/O${}_{2}$ discharge (Ar-*f* and ArO-*f*) decreased from 99${}^{\circ }$ to 75${}^{\circ }$. WCAs of the surfaces treated in the Ar/N${}_{2}$ plasma (samples ArN-f,fo) were the lowest of the three tested gas feeds with the values of 67${}^{\circ }$. The decrease in WCAs was likely caused by the addition of nitrogen functionalities as the WCAs decrease with increasing nitrogen content (Figure 5b,c). The samples treated at 22 mm/s (denoted by an *s*) became slightly smoother after the modification (Table 2). The decrease in roughness was assigned to a combination of the plasma thermal effects and LMWOMs preference to agglomerate in craters. The lowest value of WCA (36±4)${}^{\circ }$ was measured at the PP surface with the highest concentration of functional groups (sample Ar-*s*). Although the surface of the ArN-*s* sample contained a higher number of functional groups, both the ArN-*s* and ArO-*s* samples had the same WCA. Nitrogen functional groups are hydrophilic (although not as much as oxygen functionalities), and a correlation between high nitrogen content and the low WCA was also already shown for higher treatment speed (Figure 5b,c). Therefore, the higher WCA value must have been caused by the changes in the surface structure not observable by confocal microscopy (Figure 6a). Kehrer et al. [17] has recently reported a higher increase in nanoroughness (measured by AFM, the field of view 3 μm) for the PP surface treated in the N${}_{2}$ arc-based cold atmospheric plasma jet than for the same jet utilizing air as a process gas. Our Ar/N${}_{2}$ PSJ may have a similar effect resulting in the higher final WCA.

Both the components of the SFE have increased for all tested plasma treatment conditions (Figure 4d). The polar component values varied from the minimum of (0.3±0.2) mJ/m${}^{2}$, the reference, up to (25±4) mJ/m${}^{2}$, sample Ar-*f*. The trend polar SFE values are inverted to the one of WCAs; the lowest WCA corresponded to the highest polar part of the SFE. In the plasma-treated synthetic polymers, the polar part of the SFE is primarily connected to the amount of newly attached polar functional groups (hydroxyl, carboxyl, amines). Thus, the same conclusions used to explain WCA results are also valid for the polar component values. Regardless of the treatment conditions, the value of the dispersive part of the SFE increased from 33 to 37 mJ/m${}^{2}$. The stronger dispersive interaction (induced dipole–induced dipole interaction) between a non-polar liquid and the PP surface was caused by a decrease in molecular orientation in the macrostructure of polypropylene surface (cross-linking, functional group attachment, chain scission, etc.) [35].

**Figure 7:**
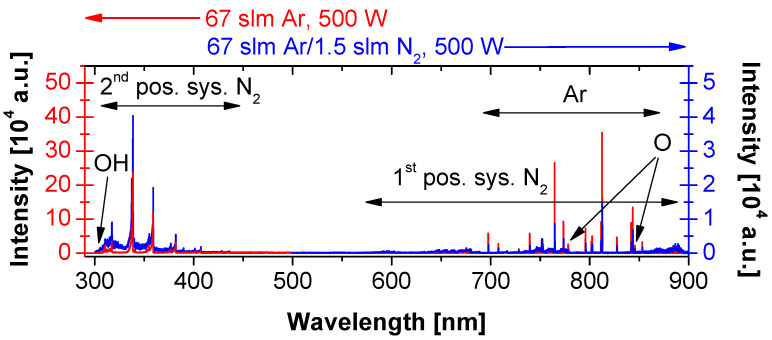
Optical emission spectra of PSJ obtained for Ar and Ar/N${}_{2}$ gas feeds at the slit exit region. The intensity of Ar discharge emission (left vertical axis) is by one order of magnitude higher than the intensity of Ar/N${}_{2}$ plasma (right vertical axis).

Knowledge of a plasma gas phase can bring insight into the processes happening on the treated PP surface. PSJ operated in an open atmosphere of humid air that intermixed into the active plasma. The optical emission spectra obtained from the region just below the slit exit confirmed the presence of O_2_, N_2_, H_2_O impurities (Figure 7). In the pure Ar discharge, the most prominent emission lines were from Ar at 690–870 nm, followed by the bands of the second positive system (SPS) of nitrogen molecules, N${}_{2}$ (C ${}^{3}\Pi_{u}$–B ${}^{3}\Pi_{g}$) transition. At the UV part of the emission spectrum, N${}_{2}$ (C ${}^{3}\Pi_{u}$–B ${}^{3}\Pi_{g}$) bands, (1–0) 315.9 nm and (2–1) 313.6 nm, partially overlapped with the relatively weak OH (A ${}^{2}\Sigma$–X ${}^{2}\Pi$) bands at 306–310 nm. Among the Ar lines in the VIS part of the spectrum, low-intensity O triplets were present at 777 nm, and 844 nm. The addition of N${}_{2}$ into the gas feed lowered emission spectrum intensity by approximately an order of magnitude. The intensity drop was most likely caused by a lower electron density reducing incidence of all the electron excitation processes [29]. All the lines reported for pure Ar were also observed in the Ar/N${}_{2}$ PSJ spectrum. Additionally, the first positive system (FPS) of nitrogen, N${}_{2}$ (B ${}^{3}\Pi_{g}$–A ${}^{3}\Sigma_{u}^{+}$), appeared at 550–900 nm. These emission bands are typical for the orange postdischarge (nitrogen afterglow) [29], a region rich with nitrogen atoms [31].

We analyzed the abundance of excited species present in both the Ar and Ar/N${}_{2}$ plasma by comparing their intensities to the Ar 811.53 nm line. In the Ar PSJ, the relative intensities of O, N${}_{2}$(C ${}^{3}\Pi_{u}$–B ${}^{3}\Pi_{g}$) and OH (A ${}^{2}\Sigma$–X ${}^{2}\Pi$) transitions were 0.07, 0.7, and 0.07, respectively. The addition of nitrogen into PSJ increased the relative intensity of N${}_{2}$(C ${}^{3}\Pi_{u}$–B ${}^{3}\Pi_{g}$) and OH (A ${}^{2}\Sigma$–X ${}^{2}\Pi$) bands to 2.7 and 0.3, respectively. The relative intensity of O lines remained unchanged. The slight increase in the relative intensity of OH (A ${}^{2}\Sigma$–X ${}^{2}\Pi$) bands was probably not a result of a larger OH abundance. Presumably, it was caused by OH bands partially overlapping with (1–0) and (2–1) bands of the SPS of N${}_{2}$.

Focusing on the plasma treatment, PP can potentially react with O${}_{2}^{*}$, O, OH, H, and N. Out of them, PP reactivity with O and OH radicals is significantly higher than with the rest [26]. The nearly unchanged relative intensity of O and OH emission lines explains the predominant number of oxygen functional groups on all treated PP surfaces (Table 2). It also explains why the reaction between mid-chain alkyl radicals and N atoms (1) is not prevalent. O and OH radicals are much more reactive, and even their lower numbers can overwhelm the larger amount of N atoms. The nitrogen functional group chemistry is driven by neutral nitrogen atoms. This plasma species cannot be directly observed in OES as the nitrogen excitation energy (14.5 eV) is too high for our type of discharge. Indirectly, for the Ar/N${}_{2}$ PSJ, the presence of the afterglow indicates high N atoms concentrations that are nicely reflected by the higher N content on the treated PP surfaces.

### 3.2. Aging of Plasma-Treated PP

The plasma treatment of polymers is not a permanent process. It is subject to post-reactions resulting in a decrease in hydrophilicity, also called aging. The time frame of aging and its severance depends on the number of functional groups and the vertical chemical gradient they form. Steeper gradients result in more pronounced changes (results of migration, diffusion, and reorientation of polar groups) as the surface tends to relax into a thermal equilibrium state. The process is called hydrophobic recovery [36,37]. The preparation of PP-epoxy-Al joints was carried out 24 h after the plasma treatment. Therefore, it is important to know if and how the treated PP surface changed after this time frame.

**Figure 8:**
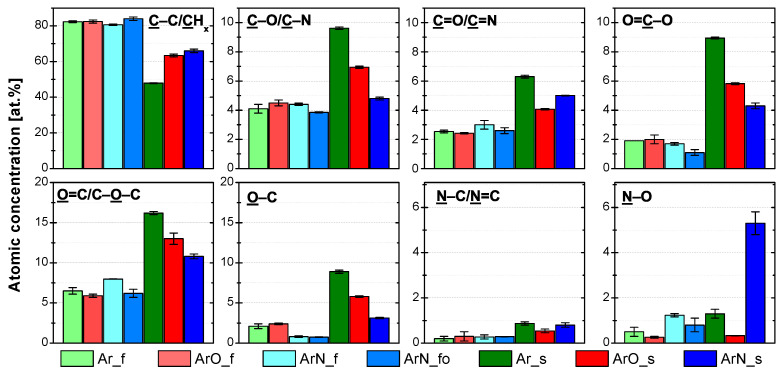
XPS C 1s, O 1s, and N 1s high-resolution spectrum components and their atomic concentrations for the plasma-treated PP samples. Spectra were measured 24 h after plasma treatment.

After 24 h, the overall concentration of functional groups (Figure 8) remained either unchanged (samples modified at 100 mm/s with two passes) or decreased by 3–5 at.% (samples treated at 22 mm/s with ten passes). Of the C 1s environments, the concentration of ester/carboxyl groups (O=C–O) terminating the PP chain [26] decreased the most. In the O 1s XPS spectrum, this decrease corresponds to a lower concentration of O=C environment. The effect of aging is the most pronounced in regards to nitrogen functionalities, as most of the amine/imine (N–C/N=C) groups oxidized during the first 24 h. Oxidation could be initiated by atmospheric oxygen, water vapor, or by oxygen already attached to PP chains explaining the decrease in the concentration of O=C–O groups. WCA is sensitive only to the topmost layer of the PP surface (compared to the 3–5 nm information depth of XPS). Therefore, WCA (Figure 4b) and SFE (Figure 4d) values reflect hydrophobic recovery the most. The reorientation of polar groups resulted in an increase in WCAs by 5${}^{\circ }$ (Ar-*f*)–27${}^{\circ }$ (Ar-*s*) and a decrease in the polar component of SFE by 2 (Ar-*f* and ArO-*f*)–12 (Ar-*s*) mJ/m${}^{2}$. The dispersive part of the SFE remained unchanged. Generally, samples treated for a longer time (denoted *s*) with the higher initial amount of functional groups and lower contact angles showed greater hydrophobic recovery, as the initial gradient of functional groups was quite steep. Outside the oxidation of part of nitrogen functionalities, changes in the samples modified at 100 mm/s with two passes were minimal.

### 3.3. Presence of Low Molecular Weight Oxidized Materials

Most of the plasma-induced functional groups exist at the top of the polymer in the form of low molecular weight oxidized materials (LMWOMs), which are shorter molecular chains created by ablation of a polymer through chain scissions [17,38]. In the C 1s high-resolution XPS spectrum, the LMWOMs’ presence can be indicated by the large concentration of ester/carboxyl groups (O=C–O) terminating the PP chain [26]. As the LMWOMs are water-soluble, the much easier and straightforward method of identifying their presence is a water-washing test. The comparison of WCAs of water-washed and unwashed samples confirmed LMWOMs’ presence (Figure 4c). WCAs increased from their initial values (Figure 4a) to 78–88${}^{\circ }$, which is still lower than the WCA of pure PP (99±2)${}^{\circ }$. Inversely, the values of the polar component of the SFE dropped to 2–5 mJ/m${}^{2}$ (Figure 4f). This suggests that not all functionalized materials exist in the form of LMWOMs. A part of the functional groups must be attached to non-soluble molecular chains of medium or high length that remained on PP surface even after water washing. The dispersive part of the SFE remained unchanged, indicating that the effect of the plasma treatment on the molecular orientation was not limited only to the topmost LMWOM layer but the PP macrostructure situated deeper into the bulk was affected as well.

**Figure 9:**
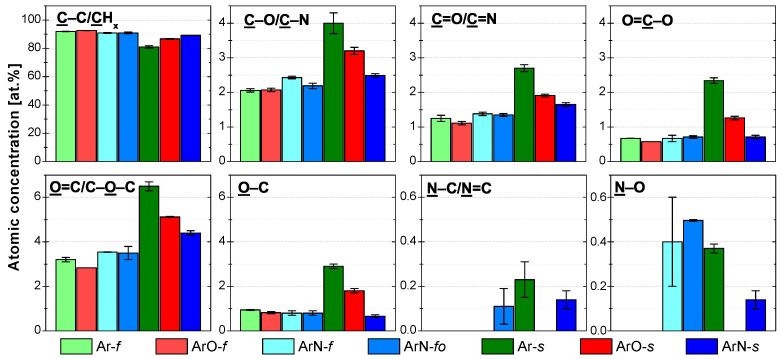
XPS C 1s, O 1s, and N 1s high-resolution spectrum components and their atomic concentrations for the water-washed plasma-treated PP samples. Spectra were measured right after washing.

For all the samples, the water-washing resulted in a major decrease in concentrations of all functional groups (Figure 9) as the soluble highly functionalized LMWOMs were washed away. Carbon content increased from the initial values (Table 2) to 90–96 at.% while oxygen content decreased to 4–9 at.%. Nitrogen content was affected by water washing the most. Its concentration either decreased to the XPS detection limit (0.5–1 at.%) or it could not be detected at all (samples with the low initial nitrogen concentration Ar-*f*, ArO-*f*, and ArO-*s*). Out of all oxygen functional groups, the amount of the ester/carboxyl groups (O=C–O) decreased the most by 60–90%. This was expected, as the O=C–O groups terminate the PP chain; therefore, most of their population is a part of the short LMWOMs chains. The concentration of the C–O/C–N and C=O/C=N populations was halved after water washing. Both O 1s environments decreased by similar amounts, by 60–90% depending on the sample. In the N 1s spectrum, the concentration of amine/imine (N–C/N=C) was lowered by more than 85% in all samples. It shows that these groups are a part of LMWOMs, most likely as the amide groups at the end of PP chains. The chemical reactions (2)–(4) resulting in the creation of amides were, together with further proofs, discussed in Section 3.1. The small amount of the N–C/N=C remaining at the PP surface was hydrolyzed by water. As a result, the percentage decrease in N–O concentration was smaller. The roughness after water washing remained the same as the roughness of the plasma-treated surfaces. For some samples, damage to the PP surface inflicted by the plasma was unveiled after the washing. However, the occurrence was random and not connected to certain treatment conditions. Water washing not influencing the roughness suggests that surface craters were filled mostly by medium-length PP chains. Overall, the results confirm the presence of LMWOMs at all treated PP surfaces. The functional group losses were higher for the samples treated at the 22 mm/s with ten passes (denoted with an *s*), pointing to a generation of a larger amount of LMWOMs.

### 3.4. Adhesion

The present study aimed to provide insight into the mechanism of the adhesive joint between the plasma-treated PP and aluminum. Several adhesives used to create high-strength joints with synthetic polymers are available on the market, but their prices are several times higher than standard epoxy glues. Plasma treatment can allow the usage of low-cost epoxy glues such as the selected epoxy adhesive DP 190 (3M) that has low performance in the joints with synthetic polymers. However, it is necessary to optimize treatment conditions or, even better, to understand the effects that can improve or deteriorate adhesive strength.

The tensile strength of the PP-Al adhesive joint after non-optimized treatment at the 100 mm/s was about three times higher (3.5–4.8 MPa) than for the untreated PP sample (1.4 MPa), as shown in Table 2. By optimizing the conditions for the Ar/N${}_{2}$ gas mixture, we were able to increase the tensile strength of the joints up to 6 MPa (Figure 5a). Out of the three elements present at the treated PP surfaces (C, O, and N), only N atomic percentages followed any dependency; in Figure 5c, we can observe how N content increased with higher nitrogen flow rate. In comparison with tensile strength results (Figure 5a), we can observe a correlation between increased N content and higher tensile strength. The WCAs (Figure 5b) follow the opposite trend relative to N content and tensile strength. this means that at the selected treatment conditions (Ar/N${}_{2}$ gas mixture, 100 mm/s movement speed, two passes), the WCA measurement can be used as a fast method to roughly determine possible adhesion qualities. The correlation between higher nitrogen content and higher tensile strength is connected to the curing process. In the DP 190 adhesive, the epoxy resin is cured by a hardener based on modified amines. The nitrogen groups present on the modified PP surface most likely behaved similarly to the amine groups of the hardener, improving the final adhesion between the adhesive and the PP. Another factor that influenced joint strengths was the applied RF power of the discharge. Higher tensile strengths were observed for samples treated at higher powers, 500 or 600 W (Figure 5a), meaning both the gas temperature and the number of reactive species were most likely higher. The increase in gas temperature might have promoted a higher degree of melting of the naturally degraded topmost PP layer, improving its bonding to the bulk.

**Figure 10:**
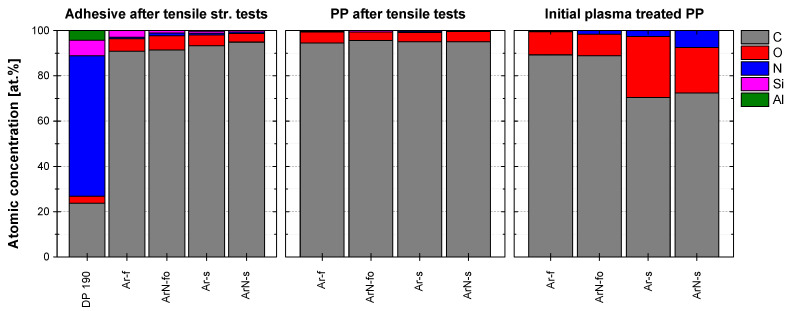
Elemental composition of the severed adhesive and PP surfaces measured after the tensile strength tests and of the initially unbounded plasma treated PP.

Decreasing the treatment speed from 100 to 22 mm/s and increasing the number of passes from two to ten did not improve the joints strength further even though the number of the functional groups attached to the modified PP surface was significantly higher (Table 2). For both tested treatment speeds, the XPS analysis of the severed joints revealed the presence of a compact PP layer on the epoxy adhesive (Figure 10). All joints, therefore, failed cohesively inside PP. Strobel et al. [19] showed that LMWOMs presence can both positively and negatively impact adhesion. If LMWOMs can be incorporated into the adhesive, LMWOMs act as adhesion enhancers. If not, a weak boundary layer of LMWOMs between the adherent and polymer is created, inhibiting strong adhesion. As the amount of functional groups present on the severed PP surface (5–7 at.%) is similar to their concentration after water washing (4–9 at.%), a weak boundary layer is likely our case. The difference between the tensile strength results for 100 and 22 mm/s movement speed (Table 2) could be explained by a variation in LMWOM layer thickness. At the lower treatment speed 100 mm/s, LMWOM layer thickness should be low (<10 nm). As a result, a large part of the LMWOMs could be incorporated into the adhesive, leaving the boundary layer thin (at some places perhaps even non-existent) and better connected to both the PP and the adherent. The confocal microscope images (Figure 6b) support the idea of a thin boundary LMWOMs layer as the PP surface after the tensile strength test maintained the typical globular structure created by the PP granules. The difference in the globules area between the plasma-treated PP before and after the tensile strength test is not a result of experiments but is caused by a variance in the initial PP sheets surfaces. The surface of PP treated at 22 mm/s with ten passes should contain higher amounts LMWOMs, and the boundary layer should be much thicker. For such samples, tensile strength appears to be limited by cohesive forces between LMWOMs as the surface of PP after the tensile strength test is flatter without the globular shapes typical for both the reference and plasma-treated and water-washed samples (Figure 6a,c might be limited only by the adhesion between the LMWOMs and the PP bulk. The interface between adhesive and LMWOM layer should be further away from PP bulk and play no role. The negative effect of the higher LMWOMs layer thickness is reflected by the number of functional groups on the surface of PP remaining at the adhesive surface. The number was higher for samples treated at 100 mm/s (C ∼ 91 at%) than at 22 mm/s (C ∼ 95 at%).

For RF PSJ, we identified two main parameters influencing adhesion results, N content and amount of LMWOMs. The highest tensile strength (6.3±0.4) MPa was achieved for the PP sample with high N content and a reasonable amount of LMWOMs. This tensile strength value is lower than what we achieved in our previous article [21], in which we utilized a different type of atmospheric pressure plasma discharge and a gliding arc jet with a side gas inlet. For the movement speed of 100 mm/s and two passes, the maximum tensile strength (9.1±0.9) MPa corresponds to elemental surface composition C:O:N = 71:24:5. The concentration of carboxyl/ethers (O=C–O) groups, signifying the presence of LMWOMs, was 7.4 at.%. These XPS results are equal to the values achieved for the PP treated by PSJ at 22 mm/s with two passes (Table 2). The PP treated in the gliding arc jet exhibited higher tensile strength that was twice as high. According to these results, a third important parameter must be influencing adhesion. We identified this parameter as plasma gas temperature. Although the measured rotational temperature of the gliding arc jet (4200 K) does not correspond to its gas temperature, it is still safe to assume that the gliding arc jet is hotter than RF PSJ ($T_{\mathrm{rot}}<1000$ K). During gliding arc jet treatment, the plasma not only creates new functional groups and scissions the PP chains but also slightly melts the topmost polymer layer. The heating of PP results in a higher degree of PP chains cross-linking and, more importantly, fuses LMWOMs and PP bulk. As a result, a weak boundary layer of LMWOMs does not form, and the adhesive joint is much stronger.

## 4. Conclusions

The RF plasma slit jet developed in the width of 150 mm was applied to the polypropylene treatment in Ar, Ar/O${}_{2}$, and Ar/N${}_{2}$. The aim was to increase PP wettability and chemical reactivity towards epoxy adhesive DP 190 bonds used to create PP-Al joints. Concerning the created functional group composition, Ar and Ar/O${}_{2}$ discharges behaved similarly. Notable changes, mainly regarding nitrogen content, were observed when using Ar/N${}_{2}$ PSJ. The highest sum of functional groups was achieved using pure Ar PSJ, a discharge with the highest electron density. Ar/O${}_{2}$ PSJ was the least effective of the three. Due to the electronegative character of oxygen plasma, electrons sustaining the discharge were lost in attachment processes, resulting in a decrease in the concentration of oxygen and nitrogen gas reactive species that initiate chemical reactions on the PP surface. The treatment in the N atoms-rich Ar/N${}_{2}$ discharge resulted in higher concentrations of nitrogen functional groups. The water-washing test showed that the nitrogen functionalities were a part of LMWOMs in the form of terminating amides groups (O=C–N) partly substituting the carboxyl/ester (O=C–O) groups.

The treatment increased the tensile strength of the adhesive bond in the range of 2.5 to 4.5 times. The highest strength was achieved using PP treated in the optimized conditions for Ar/N${}_{2}$ gas feed and the higher movement speed of 100 mm/s with two passes. All the analyzed PP-Al’s joints failed cohesively inside the PP. At 100 mm/s, failure occurred on the interface between the PP bulk and LMWOMs layer. At lower treatment speeds (22 mm/s), the joint failed directly in the LMWOMs, explaining why the high number of attached functionalities did not result in excellent adhesion. In addition to the LMWOMs, two other adhesion influencing parameters were newly identified: N content and plasma gas temperature. If the amount of LMWOMs is kept at the similar level, higher nitrogen content promotes higher adhesion between epoxy adhesive DP 190 and PP by taking part in the curing process. The plasma gas temperature impacted adhesion by affecting the weak boundary layer of LMWOMs. If the plasma gas temperature was suitably high (translational discharges), the plasma slightly melted the topmost PP layer, promoting cross-linking of polymer chains and bonding it better to PP bulk.

## Figures and Tables

**Table 1:** PP samples treated by RF plasma slit jet used in the gas feed comparison: $Q_{\mathrm{Ar}}$ denotes Ar flow rate, $Q_{x}$ is the flow rate of the additive gas (oxygen, nitrogen), *P* is the RF power, *v* is the treatment speed, and *d* is the distance between the slit exit and the sample surface. The letters *f* or *s* in the sample name denote a faster or slower treatment speed, respectively, and fo is used for the optimized conditions at the faster treatment speed. Samples were treated two or ten times (passes).

Sample	Gas Feed	$Q_{\mathrm{Ar}}/Q_{x}$ (slm)	*P* (W)	*v* (mm/s)	Passes	*d* (mm)
Ar-*f*	Ar	67	500	100	2	10
ArO-*f*	Ar/O${}_{2}$	67/1.0	500	100	2	10
ArN-*f*	Ar/N${}_{2}$	67/1.5	500	100	2	10
ArN-fo	Ar/N${}_{2}$	100/3.5	600	100	2	10
Ar-*s*	Ar	67	500	22	10	5
ArO-*s*	Ar/O${}_{2}$	67/1.0	500	22	10	5
ArN-*s*	Ar/N${}_{2}$	67/1.2	400	22	10	5

**Table 2:** Changes in the surface chemistry of plasma-treated PP samples characterized by water contact angles of a sessile drop, average atomic concentrations (without H), differences in the surface roughness measured before and after the treatment, and by tensile strengths derived from the single lap joint shear test. The error of the atomic concentration measurement was ∼1 at.%. The conditions used for treating the samples are shown in Table 1.

	WCA	C	O	N	Roughness	Tensile Str.
Sample	(${}^{\circ }$)	(at.%)	(at.%)	(at.%)	Diff. (nm)	(MPa)
Ref	99 ± 1	100				1.4 ± 0.1
Ar-*f*	74 ± 2	89	10	0.5	−1 ± 1	3.5 ± 0.3
ArO-*f*	76 ± 1	88	11	0.6	−1 ± 1	4.8 ± 0.3
ArN-*f*	67 ± 1	88	11	1.4	−1 ± 1	4.3 ± 0.3
ArN-fo	67 ± 1	89	10	1.6	2 ± 2	6.3 ± 0.4
Ar-*s*	36 ± 4	70	27	3	−5 ± 2	3.8 ± 0.2
ArO-*s*	58 ± 2	77	22	1	−4 ± 1	-
ArN-*s*	59 ± 3	72	20	8	−3 ± 1	4.5 ± 0.2

## Data Availability

The data presented in this study are available in the main text.
